# Safety assessment and antioxidant activity of Lantana montevidensis leaves: Contribution to its phytochemical and pharmacological activity

**DOI:** 10.17179/excli2017-163

**Published:** 2017-04-21

**Authors:** Luiz Marivando Barros, Antonia Eliene Duarte, Emily Pansera Waczuk, Katiane Roversi, Francisco Assis Bezerra da Cunha, Mirian Rolon, Cathia Coronel, Maria Celeste Vega Gomez, Irwin Rose Alencar de Menezes, José Galberto Martins da Costa, Aline Augusti Boligon, Waseem Hassan, Diogo Onofre Souza, João Batista Teixeira da Rocha, Jean Paul Kamdem

**Affiliations:** 1 Universidade Regional do Cariri-URCA, Centro de Ciências Biológicas e da Saúde-CCBS, Departamento de Ciências Biológicas, CEP: 63105-000, Pimenta, Crato-Ceará, Brazil; 2 Departamento de Bioquímica e Biologia Molecular, Programa de Pós-Graduação em Bioquímica Toxicológica, Universidade Federal de Santa Maria, Santa Maria, RS 97105-900, Brazil; 3 Departamento de Farmacologia, Programa de Pós-Graduação em Ciências Farmacêuticas, Farmatox, Universidade Federal de Santa Maria, Santa Maria, RS 97105-900, Brazil; 4 Centro Para El Desarrollo de La Investigación Científica (CEDIC),Fundación Moisés Bertoni /Laboratorios Diaz Gill, Asuncion, Paraguay; 5 Universidade Regional do Cariri-URCA, Departamento de Química Biológica, Laboratório de Farmacologia, Crato-Ceará, Brazil; 6 Universidade Regional do Cariri-URCA, Departamento de Química Biológica, Laboratório de Pesquisas de Produtos Naturais, CEP 63.105.000, Crato-Ceará, Brazil; 7 Laboratório de Fitoquímica, Departamento de Farmácia Industrial, Universidade Federal de Santa Maria, Santa Maria, RS 97105-900, Brazil; 8 Institute of Chemical Sciences, University of Peshawar, Peshawar -25120, Khyber Pakhtunkhwa, Pakistan; 9 Departamento de Bioquímica, Instituto de Ciências Básica da Saúde, Universidade Federal do Rio Grande do Sul, Porto Alegre, RS CEP 90035-003, Brazil

**Keywords:** Lantana montevidensis, genotoxicity, cytotoxicity, osmotic fragility, HPLC-DAD

## Abstract

*Lantana camara,* the widely studied species, and *L. montevidensis,* the less studied species of the genus *Lantana* are both used in traditional medicine for the same purpose (anti-asthma, anti-ulcer, anti-tumor, etc). However, little is known about the toxicity of *L. montevidensis* and there is limited information on its chemical constituents. Here, we investigated for the first time the genotoxicity and cytotoxicity of the ethanolic (EtOH) and aqueous extracts from the leaves of *Lantana montevidensis* in human leukocytes, as well as their possible interaction with human erythrocyte membranes *in vitro*. The antioxidant activities of both extracts were also investigated in chemical and biological models. Treatment of leukocytes with EtOH or aqueous extracts (1-480 µg/mL) did not affect DNA damage index, but promoted cytotoxicity at higher concentrations (240-480 µg/mL). Both extracts did not modify the osmotic fragility of human erythrocytes. The extracts scavenged DPPH radical and prevented Fe^2+^-induced lipid peroxidation in rat's brain and liver homogenates, and this was likely not attributed to Fe (II) chelation. The HPLC analysis of the extracts showed different amounts of polyphenolic compounds (isoquercitrin, gallic acid, catechin, ellagic acid, apigenin, kaempferol, caffeic acid, rutin, quercitrin, quercetin, chlorogenic acid, luteolin) that may have contributed to these effects. These results supported information on the functional use of *L. montevidensis* in folk medicine.

## Introduction

Plants with therapeutic actions have been used by rural communities and even urban population in the treatment of several diseases, and their beneficial effects have been attributed to their chemical constituents. This is at least in part, because their chemical constituents may have antioxidant activity, thereby, preventing the oxidative damage resulting from oxidative stress, which has been implicated in many diseases including stroke, cancer and other neurodegenerative diseases (Uttara et al., 2009[37]; Visconti and Grieco 2009[38]; Rodrigo et al., 2013[30]; Kamdem et al., 2016[18]). 

The genus *Lantana* is composed of about 150 species (Ghisalbert, 2000[12]) in which *L. camara* and *L. montevidensis* are represented. The two species are very similar from a botanical point of view and are used in popular medicine as carminative, antispasmodic, antiemetic and to treat respiratory infections, rheumatism, wounds, asthma, biliary fever, bronchitis, tumor, ulcers, high blood pressure, among others (Barreto et al., 2010[3]; Kalita et al., 2012[17]). 

Although, *L. camara* is well studied for its pharmacological properties (Kalita et al., 2012[17]), it is recognized so far among the most toxic plants (Ghisalbert, 2000[12]; Kalita et al., 2012[17]). Its toxic effects to human and livestock has been reported in Australia, India, New Zealand, South Africa and America, and were attributed to the presence of lantadenes A, B and D (Kalita et al., 2012[17]). In addition, the essential oil obtained from the leaves of *L. camara* was recently found to be toxic to NCTC929 fibroblasts (Barros et al., 2016[4]).

*L. montevidensis* (Family, Verbenaceae), popularly known as “camará” or “cambará”, is a sprawling shrub native to Brazil and Uruguay. It is considered an invasive species in many parts of the world where it has been introduced as an ornamental plant (Taylor, 2015[36]). The dried leaves of *L. montevidensis* prepared in different ways (infusion, maceration, decoction), and its essential oil have been traditionally used for the same purposes like *L. camara* (Hashimoto, 1996[15]). In opposition to the widely studied *L. camara*, very few studies have demonstrated the pharmacological potential of *L. montevidensis*. Extracts and essential oil from the leaves of *L. montevidensis* have been reported to exhibit antibacterial activity with the potential to modulate antibiotics drugs used in clinical infections (Barreto et al., 2010[3]; Sousa et al., 2013[35]). In addition, methanolic extract of the leaves showed anti-inflammatory, anti-pyretic, analgesic, antioxidant and antibacterial activities (Sousa et al., 2015[34]). Phytochemical studies revealed that the flavonoid fraction from the leaves of *L. montevidensis* exhibits antiproliferative activity against human gastric adenocarcinoma, human uterine carcinoma and murine melanoma cell lines (Nagão et al., 2002[26]). Triterpenoids and flavones are likely to be the major components of *L. montevidensis* leaves extracts (Makboul et al., 2013[23]), while β-caryophyllene, germacrene and bicyclogermacrene are the main constituents of its essential oil (Sousa et al., 2013[35]). 

Considering the lack of information in the literature regarding the biological activities of *L. montevidensis*, and its chemical composition, the present study aimed to chemically characterize the aqueous and ethanolic extracts from the leaves of *L. montevidensis*, evaluate their bioactive properties. Their potential antioxidant (DPPH, TBARS, and Fe^2+^ chelation) activities, toxic effects (genotoxicity and cytotoxicity), as well as the influence on osmotic fragility were also investigated in human leukocytes and erythrocytes respectively.

## Materials and Methods

### Chemicals

All chemicals were of analytical grade. Acetonitrile, phosphoric acid, gallic acid, caffeic acid, ellagic acid, chlorogenic acid and catechin were purchased from Merck (Darmstadt, Germany). Quercetin, quercitrin, isoquercitrin, kaempferol, luteolin, apigenin and rutin were acquired from Sigma Chemical Co. (St. Louis, MO, USA). High performance liquid chromatography (HPLC-DAD) was performed with a Shimadzu Prominence Auto Sampler (SIL-20A) HPLC system (Shimadzu, Kyoto, Japan), equipped with Shimadzu LC-20AT reciprocating pumps connected to a DGU 20A5 degasser with a CBM 20A integrator, SPD-M20A diode array detector and LC solution 1.22 SP1 software. 1,1-diphenyl-2-picrylhydrazyl (DPPH), ascorbic acid, malonaldehydebis-(dimethylacetal) (MDA), thiobarbituric acid (TBA), sodium azide and hydrogen peroxide (H_2_O_2_) were obtained from Sigma Chemical Co. (St. Louis, MO, USA).

### Plant material 

The leaves of *Lantana montevidensis* were collected in Padre Cicero, Crato - Ceara (7^◦^ 22´S; 39^◦^ 28´W, 492 m above sea level), Brazil. The plant material was identified and the specimen deposited in the Herbarium Caririense Dárdano de Andrade - Lima, Regional University of Cariri (URCA), with voucher number 7519. The aqueous extract of *L. montevidensis *was prepared by crushing 300 g of fresh leaves, mixed with 2 L of hot water and the mixture was allowed to stand for 3 days. While the ethanolic extract (EtOH) was prepared by crushing 300 g of fresh leaves and macerating in 1.5 L of 92 % ethanol, this was also allowed to stand for 3 days. On the 3^rd^ day, the mixture was filtered and the filtrate evaporated under reduced pressure and lyophilized to obtain a dark brown solid. The prepared extracts (aqueous and ethanolic) were stored in the freezer until being used. 

### Characterization of aqueous and ethanolic extracts from the leaves of L. montevidensis by HPLC-DAD

Reverse phase chromatographic analysis was carried out under gradient conditions using Phenomenex C_18 _column (4.6 mm x 250 mm) packed with 5 μm diameter particles; the mobile phase was: (A) acetonitrile: water (95:5, v/v) and (B) water: phosphoric acid (98:2, v/v), and the composition gradient was: 5 % of B until 10 min and changed to obtain 20 %, 40 %, 60 %, 70 % and 100 % B at 20, 30, 40, 50 and 60 min, respectively, following the method described by Colpo et al. (2014[8]) with slight modifications. The ethanolic and aqueous extracts from the leaves of *L. montevidensis* and the mobile phase were filtered through 0.45 μm membrane filter (Millipore) and then degassed by ultrasonic bath prior to use. The extracts (ethanolic and aqueous) of *L. montevidensis* were analyzed at 30 mg/mL. The flow rate was 0.6 mL/min and the injection volume was 50 μL. Stock solutions of standard references were prepared in the HPLC mobile phase at a concentration range of 0.025 - 0.400 mg/mL for catechin, quercetin, quercitrin, isoquercitrin, kaempferol, luteolin, apigenin and rutin; and 0.030 - 0.250 mg/ml for gallic, chlorogenic, ellagic and caffeic acids. Quantification of these compounds was carried out by integration of the peaks using the external standard method, at 270 nm for gallic and ellagic acids, 280 nm for catechin, 327 nm for chlorogenic and caffeic acids, and 365 for quercetin, quercitrin, isoquercitrin, kaempferol, apigenin, luteolin and rutin. The chromatography peaks were confirmed by comparing its retention time with those of reference standards and by DAD spectra (200 to 600 nm). The calibration curve for the standards used is presented in Table 1(Tab. 1). Limit of detection (LOD) and limit of quantification (LOQ) were calculated based on the standard deviation of the responses and the slope using three independent analytical curves. LOD and LOQ were calculated as 3.3 and 10 σ/S, respectively, where σ is the standard deviation of the response and S is the slope of the calibration curve.

### Antioxidant activity in chemical and biological models

#### 1, 1-diphenyl-2-picrylhydrazyl (DPPH) radical scavenging activity

The antioxidant activity was determined by the ability of the aqueous and ethanolic extracts from the leaves of *L. montevidensis* to scavenge the DPPH radical, as described by Kamdem et al. (2012[20]) with some modifications. Briefly, 50 µL of ethanolic and aqueous extracts in different concentrations (1-480 µg/mL) were mixed with 100µL of freshly prepared DPPH solution (0.3 mM in ethanol). The plate was kept in the dark at room temperature for 30 min. The reduction of DPPH color was measured by monitoring the decrease in absorption at 517 nm using a microplate reader (SpectraMax, USA). Ascorbic acid was used as positive control. The percent DPPH inhibition was calculated as follows:

% inhibition = 100 - [100 (A_sample_ - A_blank_)/A_control_], where A_sample_ is the absorbance of the tested sample with DPPH; A_blank_, the absorbance of the test tube without the addition of DPPH and A_control_ is the absorbance of DPPH solution.

#### Fe^2+ ^chelating activity of the extracts 

The Fe^2+ ^chelating activity of the ethanolic and aqueous extracts from the leaves of *L. montevidensis* was determined using a modified method of Kamdem et al. (2013[19]). The reaction mixture containing 58 µL of saline solution (0.9 %, w/v), 45 µL Tris-HCl (0.1 M, pH, 7.5), 27 µL of extracts (1-60 µg/mL) and 36 µL of 110 µM FeSO_4_ was incubated for 10 min at 37 °C. Subsequently, 34 µL of 1,10-phenanthroline (0.25 %, w/v) was added and the absorbance of the orange coloured complex formed was measured at 0, 10 and 20 min at 510 nm (against blank solutions of the samples) using microplate reader (SpectraMax, USA). The same procedure was performed for the control (i.e., Fe^2+^, without the extract). To ascertain the chelating potential of the extracts, we determined the potential reduction of any Fe^3+^ (that might be formed during the incubation periods) by adding the reducing agent, ascorbic acid (to give a final concentration of 5 mM) to the reaction mixture. The absorbance was then determined after 5, 10 and 20 min following ascorbic acid addition. This is because the extracts could be oxidizing Fe^2+^ to Fe^3+^, leading to a decrease in absorbance that was not related to Fe^2+^ chelation. Schedule for the evaluation of Fe^2+ ^chelation or oxidation by the extracts is presented in Table 2(Tab. 2). 

#### Thiobarbituric acid reactive substances (TBARS) assay

TBARS production was determined as described by Ohkawa et al. (1979[27]) and modified by Barbosa-Filho et al. (2014[2]). The rats were killed by decapitation and the whole brain and liver were rapidly removed, weighed and placed on ice. The tissues were immediately homogenized in cold 10mM Tris-HCl, pH 7.4 (1:10 w/v for the liver and 1:5 w/v for the brain) and centrifuged at 3600 rpm for 10 min. The pellet was discarded and the supernatant was used for the assay. Aliquots of homogenates of brain or liver (20 µL) were incubated with 10 µM FeSO_4_, in the presence or absence of extracts (1-480 µg/mL) at 37 °C for 1 h to induce lipid peroxidation. Subsequently, 40 µL of sodium dodecyl sulfate (8.1 %), 100mL of acetic acid/HCl (pH 3.4) and 100 µL of 0.6 % thiobarbituric acid (TBA) were added and the reaction mixture was incubated at 100 °C for 1 h. After cooling, the samples were centrifuged for 2 min at 6000 rpm and the absorbance of the supernatant was read at 532 nm using an ELISA microplate reader (SpectraMax, USA). This work was performed in accordance with the guidelines of the Ethics Committee of the Federal University of Santa Maria (UFSM) and approved by the Ethics Committee of UFSM (076.2012-2).

#### Blood sample collection and preparation of human leukocytes and erythrocytes

The heparinized venous blood was obtained from healthy volunteer donors from the Hospital of the Federal University of Santa Maria (HUFSM), Santa Maria-RS, Brazil, (age 26 ± 9). The study was approved by the Ethics Committee of UFSM and registered under the protocol number 0089.0.243.000-07. Human leukocytes and erythrocytes were obtained as previously described (Kamdem et al., 2013[19]; Barbosa-Filho et al., 2014[2]). The leukocytes were separated by differential sedimentation rate using 5 % dextran and subsequent adjustment of samples 2 x 10^6^ leukocytes/mL with Hank's buffered saline solution (HBSS)/heparin (5.4 mM KCl, 0.3 mM Na_2_HPO_4_, 0.4 mM KH_2_PO4, 4.2 mM NaHCO_3_, 1.3 mM CaCl_2,_ 0.5 mM MgCl_2_, 0.6 mM MgSO_4_, 137 mM NaCl, 10 mM D-glucose and 10 mM Tris-HCl, heparin 15 IU/ mL, adjusted to pH 7.4). The blood samples were centrifuged at 2000 rpm for 5 min at room temperature to obtain the erythrocytes. The plasma was aspirated and the cell pellet was washed three times with phosphate buffered saline (6.1 mM, pH 7.4, containing 150 mM NaCl).

#### Determination of osmotic fragility in erythrocytes

The effect of ethanolic and aqueous extracts from the leaves of *L. montevidensis* on the osmotic fragility of human erythrocytes was estimated by measuring their resistance to hemolysis in increasing concentrations of salt solutions as described by Godal and Heistø (1981[13]) adapted by Barbosa-Filho et al. (2014[2]). Five hundred microliters (500 µL) of erythrocytes, 100 µL of various concentrations of aqueous and ethanolic extracts (1-480 µg/mL) and 900 mL of phosphate buffered saline (PBS) (6.1 mM, pH 7.4, containing 150 mM NaCl) were pre-incubated for 3 h at 37 °C. After incubation, the samples were mixed and centrifuged at 2500 rpm for 10 min and the supernatant was discarded. The erythrocytes were washed twice with PBS, centrifuged at 2500 rpm for 2 min and the supernatant discarded. Treated and untreated erythrocytes (7.5 µL) were then incubated with 1.5 mL of various concentrations of NaCl (0 to 0.9 %), pH 7.4 for 20 min. The samples were homogenized and centrifuged at 2000 rpm for 5 min. The obtained supernatant from each Eppendorf was transferred to microplate and the lysis of erythrocytes was followed by measuring the absorbance of the hemoglobin content in the supernatant at 540 nm using microplate reader (SpectraMax, USA). The results were expressed as percentage of control.

#### Evaluation of genotoxicity by the Comet assay

The potential genotoxic effect of the extracts was investigated according to the methods described by Collins (2014[7]) with some modifications. Leukocytes were isolated as described above and treated with various concentrations of aqueous and alcoholic extracts of *L. montevidensis* (1-480 µg/mL) for 3 hours. The comet assay was performed according to the following steps: (1) 15 µL of leukocyte suspension (2 x 10^6^ leukocytes/mL) was mixed with low-melting agarose and then, (2) added to 90 ml 0.75 % LMP agarose (w/v), mixed, and placed on a slide pre-coated with normal melting point agarose (1 % w/v), (3) a coverslip was added and the samples allowed to solidify at 4 °C, (4) coverslips were removed and slides were placed in a lysis solution (2.5M NaCl; 100 mM EDTA, 8 mM Tris-HCl, 1 % Triton X -100 pH 10-10.5), for 24 hours under protection from light; (5) after lysis, the slides were placed in a bucket containing neutralizing solution (400 mM Tris-HCl, pH 7.5) for 15 min; (6) then, they were incubated in electrophoresis solution (300 mM NaOH, 1 mM EDTA, pH 13.5) for 20 min at 4 °C under the condition of 25 V, 300 mA, 7W; (7) the slides were washed three times in distilled water and allowed to dry at room temperature; (8) slides were rehydrated for 3 minutes in distilled water, fixed for 10 min in 15 % trichloroacetic acid, 5 % zinc sulfate and 5 % glycerol, and then washed three times in distilled water and allowed to dry at room temperature; (9) the slides were stained with 5 % sodium carbonate, 0.1 % ammonium nitrate, 0.1 % silver nitrate, 0.25 % tungstosilicic acid and 0.15 % formaldehyde; (10) staining was stopped with 1 % acetic acid and sheets air dried; (11) finally, the slides were visualized and scored according to the tail length into five classes (from class 0: no damage, no tail; to class 4: maximum damage, comet without heads) under blind conditions for at least two individuals. DNA damage was expressed as DNA damage index (DI), which is based on the length of the migration. The DI was calculated from the cells in different damage classes as follows: DI = 1n1 + 2n2 + 3n3 + 4n4, where n1-n4 denotes the number of cells with the level 1-4 of damage. Methyl methanesulfonate (MMS) (20 µM) was used as positive control while distilled water was used as negative control. 

#### Cytotoxicity evaluation by Trypan blue dye exclusion assay

The potential cytotoxic effects of the extracts were performed using a modified method of Smith et al. (2007[32]). Briefly, 2.5 mL of the extracts (1-480 µg/mL) was added to 497.5 mL of leukocytes suspension and incubated in the presence or absence of hydrogen peroxide (2 mM) + azide (1 mM) at 37 °C for 3 hours. 50 µL of treated leukocytes was mixed with 50 µL of 0.4 % Trypan blue and allowed to stand for 5 min at room temperature. From the mixture, an aliquot of 10 µL was checked microscopically for viability using a hemocytometer. Viability was expressed as the percentage of viable cells among the total cells.

### Statistical analysis

Results are expressed as mean ± standard error of mean (SEM). One or two-way analysis of variance (ANOVA) followed by Bonferroni post-test were used where appropriated, to assess the differences between the groups. P < 0.05 was considered statistically significant. 

## Results

### Chemical constituents of ethanolic and aqueous extracts from the leaves of L. montevidensis by HPLC 

**Figure 1:**
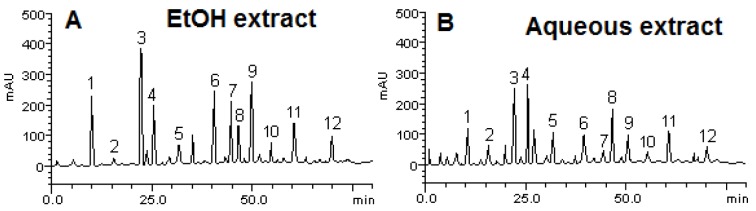
Phenolics and flavonoids constituents of ethanolic (A) and aqueous (B) extracts from the leaves of *L. montevidensis* by high performance liquid chromatography (HPLC). Gallic acid (peak 1), catechin (peak 2), chlorogenic acid (peak 3), caffeic acid (peak 4), ellagic acid (peak 5), rutin (peak 6), quercitrin (peak 7), isoquercitrin (peak 8), quercetin (peak 9), kaempferol (peak 10), luteolin (peak 11) and apigenin (peak 12). The retention time of each compound is shown in Table 1.

Phenolic acids and flavonoids determined in the ethanolic (Figure 1A(Fig. 1)) and aqueous (Figure 1B(Fig. 1)) extracts of the leaves of *L. montevidensis* are depicted in Figure 1(Fig. 1). Both extracts revealed the presence of gallic acid (R_t_ = 10.05 min, peak 1), catechin (R_t_ = 15.72 min, peak 2), chlorogenic acid (R_t_ = 22.46 min, peak 3), caffeic acid (R_t_ = 25.13 min, peak 4), ellagic acid (R_t_ = 32.09 min, peak 5), rutin (R_t_ = 39.87 min, peak 6), quercitrin (R_t_ = 44.76 min, peak 7), isoquercitrin (R_t_ = 45.61 min, peak 8), quercetin (R_t_ = 49.97 min, peak 9), kaempferol (R_t_ = 55.02 min, peak 10), luteolin (R_t_ = 60.23 min, peak 11) and apigenin (R_t_ = 70.15 min, peak 12). 

The quantitative analysis of these constituents indicate that chlorogenic acid (8.59 ± 0.01 mg/g) and quercetin (6.03 ± 0.01 mg/g) were the major constituents found in the EtOH extract, while catechin (0.48 ± 0.03 mg/g) and kaempferol (1.57 ± 0.02 mg/g) were less abundant (Table 3(Tab. 3)). Similarly, caffeic acid (6.25 ± 0.01 mg/g) and chlorogenic acid (6.17 ± 0.03 mg/g) were the major constituents found in the aqueous extract, while, apigenic (0.86 ± 0.01 mg/g) and quercitrin (0.71 ± 0.01 mg/g) were the minor constituents (Table 3(Tab. 3)).

### Antioxidant activity 

#### DPPH free radical scavenging ability

The effect of EtOH and aqueous extracts from the leaves of *L. montevidensis* on DPPH reduction is presented in Table 4(Tab. 4). Both extracts and the ascorbic acid used as reference, showed antioxidant activity against DPPH radical scavenging in a concentration- dependent manner. At the lowest concentration tested (1 µg/mL), the radical scavenging potential of the aqueous extract (24.20 ± 2.32 %) was more pronounced, even than that of ascorbic acid (9.55 ± 2.01 %). Based on the IC_50_ values, the ability to scavenge the DPPH free radical of the aqueous extract was two times higher than that of EtOH extract (108.2 ± 3.46 *vs.* 290.5 ± 1.97 µg/mL). However, ascorbic acid exhibited the highest DPPH free radical scavenging, with IC_50_ of 37.05 ± 1.69 µg/mL (Table 4(Tab. 4)).

#### Iron chelation or oxidizing effect of L. montevidensis leaf extracts

**Figure 2:**
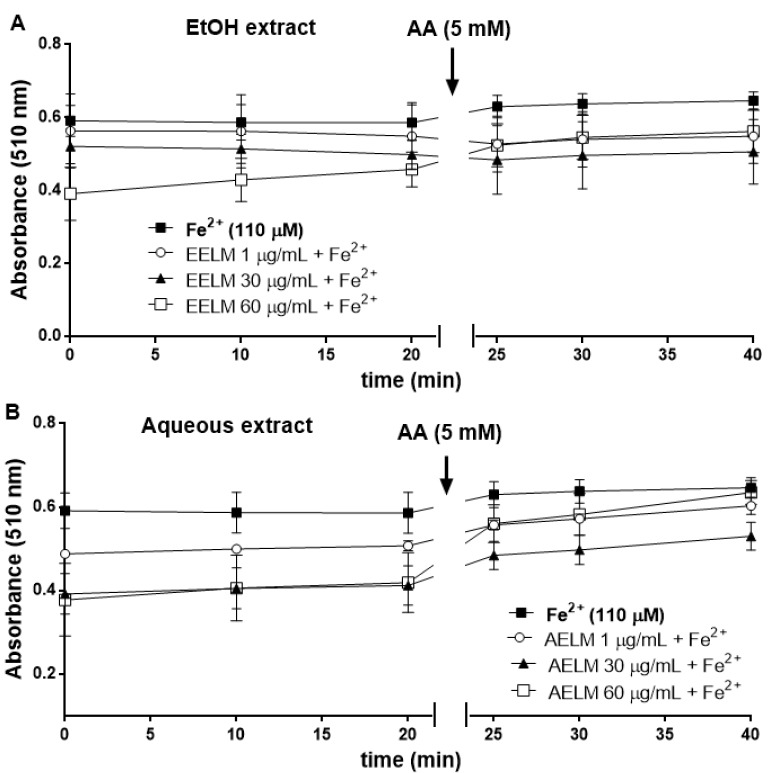
Oxidation of Fe^2+^ by EtOH (A) and aqueous (B) extracts from the leaves of *L. montevidensis*. The extracts (1-60 µg/mL) were incubated with FeSO_4_ (110 µM) for 10 min. Then, ortophenanthroline was added and the absorbance of the reaction mixture was measured at 0, 10 and 20 min following its addition. After the last reading (at 20 min), 5 mM ascorbic acid (AA) was added to the reaction mixture, and the absorbance was read again after 5 min (at 25 min), 10 min (at 30 min) and 20 min (at 40 min) (see Table 1 for details). Values represent the mean ± SEM of 3 independent experiments performed in duplicate.

In this assay, the rate of the reduction in the absorbance of the orange complex formed by Fe^2+^ and orthophenanthroline allows estimation of a co-existent chelator. As it can be seen in Figure 2A and 2B(Fig. 2), EtOH (1-60 µg/ mL) and aqueous extracts (1-60 µg/mL) caused a decrease in the absorbance at 510 nm compared with Fe^2+^. In order to investigate whether the reduction in the absorbance in the presence of extracts was attributed to Fe^2+^ chelation or oxidation, ascorbic acid (AA) was added to the reaction medium after 20 min of incubation of Fe^2+^ with the extracts and orthophenanthroline. Addition of ascorbic acid caused only a modest increase in the absorbance at 510 nm and this was more apparent for the aqueous extract (Figure 2B(Fig. 2)). The most plausible interpretation of these results is that the extracts can chelate Fe^2+^ and accelerate the oxidation of Fe^2+^ to Fe^3+^. However, this Fe^3+^ was only partially or not released from the complex; because otherwise ascorbic acid should have reduced Fe^3+^ to Fe^2+^, resulting to the formation of the complex between Fe^2+^ and orthophenanthroline. But, this occurred only to a limited extent.

#### Effect of L. montevidensis leaf extracts on lipid peroxidation (LPO) induced by iron in rat brain and liver homogenates

**Figure 3:**
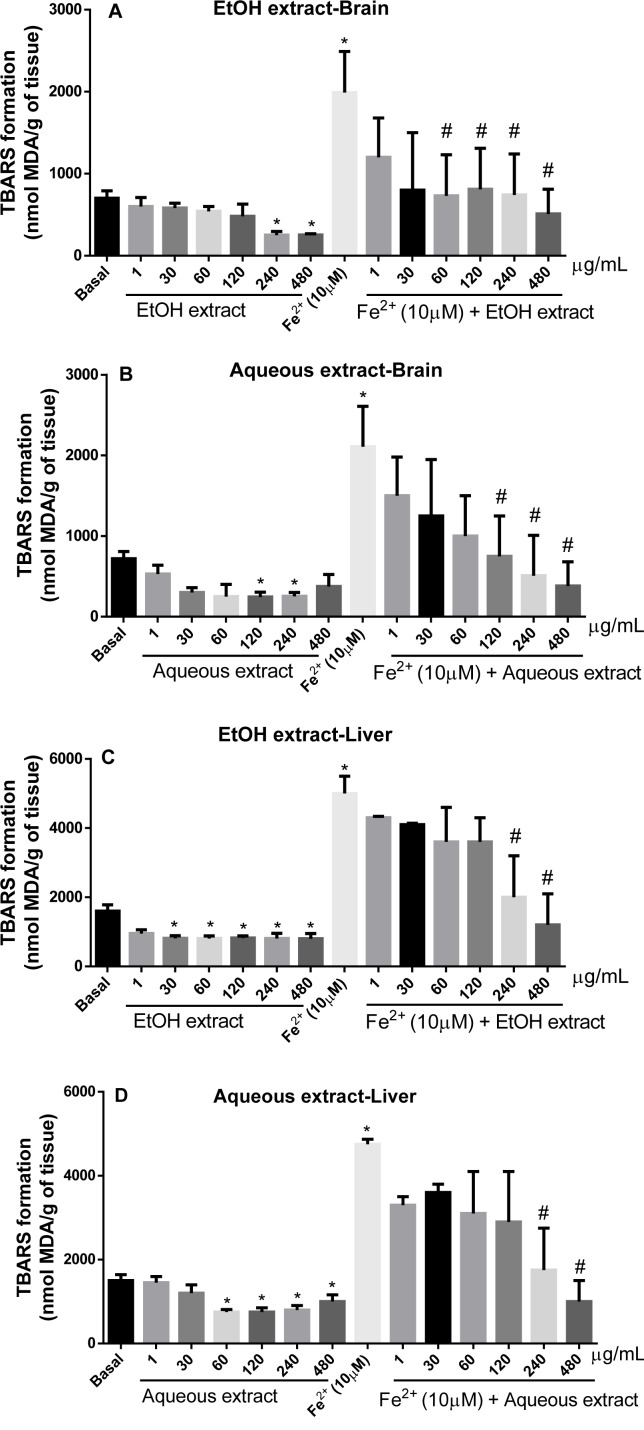
Inhibitory potential of EtOH and aqueous extracts from the leaves of *L. montevidensis* on Fe^2+^-induced lipid peroxidation in rat brain (A and B) homogenates. The results are expressed as mean ± SEM of n = 3 independent experiment performed in duplicate. *p < 0. 05 against basal; and # p < 0.05 *vs.* Fe^2+^. Inhibitory potential of EtOH and aqueous extracts from the leaves of *L. montevidensis* on Fe^2+^-induced lipid peroxidation in liver (C and D) homogenates. The results are expressed as mean ± SEM of n = 3 independent experiment performed in duplicate. *p < 0. 05 against basal; and # p < 0.05 *vs.* Fe^2+^.

In the rat brain homogenate, the potential of EtOH (Figure 3A(Fig. 3)) and aqueous (Figure 3B(Fig. 3)) extracts to inhibit LPO was assessed by the amount of MDA produced. Both extracts caused a significant reduction in the amount of MDA produced when compared to the basal (p < 0.05; Figure 3A and 3B(Fig. 3)). The basal inhibition with the EtOH extract was effective at 240 µg/mL where its maximum effect was attained (Figure 3A(Fig. 3)). Whereas, inhibition by the aqueous extract occurred at concentrations of 120 and 240 µg/mL (Figure 3B(Fig. 3)). There was a significant increase in the amount of MDA in Fe^2+ ^(10 µM)-induced LPO when compared with the basal, in rat brain homogenate (Figure 3A and 2B(Fig. 3); p < 0.05). EtOH extract (60-480 µg/mL) was effective to protect against LPO induced by Fe^2+ ^in brain homogenate (Figure 3A(Fig. 3)) when compared to iron alone. The same effect was observed in a concentration-dependent manner with the aqueous extract (120-480 µg/mL) (Figure 3B(Fig. 3)). 

The capacity of EtOH and aqueous extracts from the leaves of *L. montevidensis *to inhibit Fe^2+^-induced lipid peroxidation was also investigated in rat liver homogenate. The brain and liver were used because they have distinct enzymes that can interact with the components of the extracts and possibly change their antioxidant activities (De Freitas and Rocha, 2011[9]). 

As shown in Figure 3C(Fig. 3) and Figure 3D(Fig. 3) respectively, EtOH (30-480 µg/mL) and aqueous (60-480 µg/mL) extracts caused a significant reduction in the amount of MDA produced under basal condition, when compared with the basal (p < 0.05). As expected, Fe^2+^ (10 µM) significantly induced LPO in the rat liver homogenate, which was evidenced by the amount of MDA produced (Figure 3C and 3D(Fig. 3)). Treatment with both extracts protected against iron induced LPO in a concentration dependent manner (Figure 3C and 3D(Fig. 3)). But, the reduction in MDA produced was statistically significant only at concentrations of 240 and 480 µg/mL for both extracts (Figure 3C and 3D(Fig. 3)). 

#### Cytotoxicity and anti-cytotoxic effects of L. montevidensis leaf extracts

**Figure 4:**
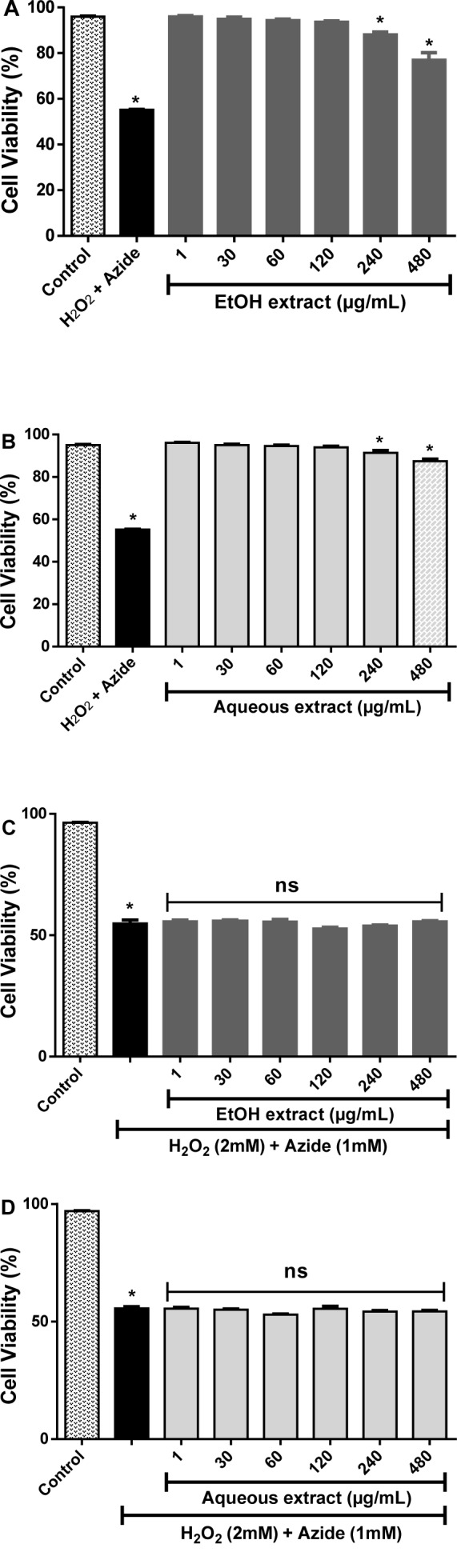
Effect of ethanolic and aqueous extracts from the leaves of *L. montevidensis* on human leukocytes in the absence (A and B) and presence (C and D) of H_2_O_2_. The results are expressed as percentage of control. Each column represents the mean ± SEM of four independent experiments. *p < 0.05 against control, ns-not significant

The potential cytotoxic effect of EtOH and aqueous extracts from the leaves of *L. montevidensis *was investigated in human leukocytes as well as their capacity to protect against H_2_O_2_ cytotoxicity. As it can be observed in Figure 4(Fig. 4), EtOH (Figure 4A(Fig. 4)) and aqueous (Figure 4B(Fig. 4)) extracts alone, caused a significant decrease in the cell viability at the higher concentrations tested (240-480 µg/ mL) when compared with the control (p < 0.05). 

We also investigated whether these extracts could protect against the exogenous oxidative stressor, H_2_O_2_ (2 mM). As expected, there was a significant decrease of cell viability (~ 41.43 %) of leukocytes treated with H_2_O_2 _when compared with the control (p < 0.05, Figure 4C and 4D(Fig. 4)). Co-treatment with EtOH (Figure 4C(Fig. 4)) or aqueous (Figure 4D(Fig. 4)) extracts did not change H_2_O_2 _cytotoxicity (p > 0.05).

#### Genotoxicity effect of L. montevidensis leaf extracts

**Figure 5:**
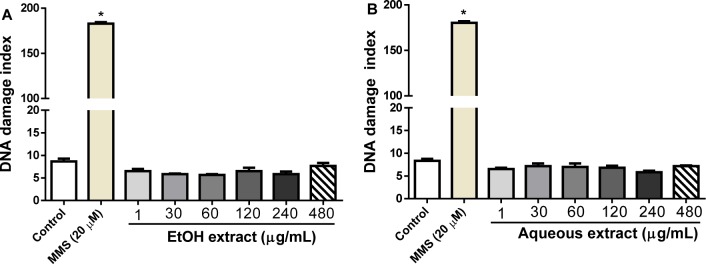
Evaluation of genotoxicity effects of ethanolic (A) and aqueous (B) extracts from the leaves of *L. montevidensis* by the Comet assay. MMS-methyl methanesulfonate. Results are mean ± SEM of three independent experiments. *p < 0.05 against control

Figure 5(Fig. 5) shows that incubation of human leukocytes with EtOH (Figure 5A(Fig. 5)) or aqueous (Figure 5B(Fig. 5)) extracts at all the concentrations tested did not have any effect on DNA damage, when compared to the control (p > 0.05). However, methyl methanesulfonate (MMS) used as positive control, dramatically increased the DNA damage index compared with the control (p < 0.05), suggesting a possible induction of DNA single-strand breaks. 

#### Effect of ethanolic and aqueous extracts on erythrocytes osmotic fragility

**Figure 6:**
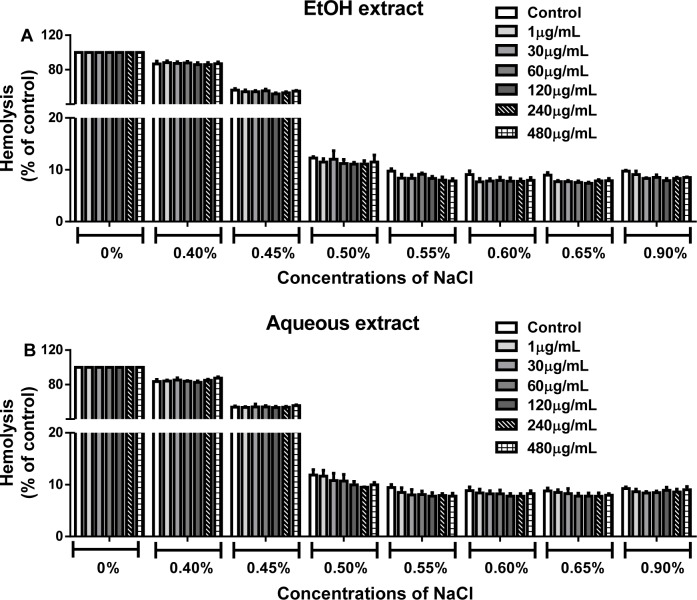
Osmotic fragility of erythrocytes treated with EtOH (A) and aqueous (B) extracts from the leaves of *L. montevidensis*. Hemolysis was expressed in percentage of the positive control (Triton-100). Treated erythrocytes were added to various concentrations of NaCl (0-0.9 %) and incubated for 20 min and the absorbance of the supernatants were measured at 540 nm. The bars represent the means of n = 3 independent experiments performed in duplicate.

Figure 6(Fig. 6) shows that there was a complete hemolysis (100 %) in the control group containing 0 % NaCl (i.e., distilled water). Treatment of human erythrocytes with EtOH (Figure 6A(Fig. 6)) or aqueous (Figure 6B(Fig. 6)) extracts (1-480 µg/mL) did not modify the percentage of erythrocyte fragility (p > 0.05) at different salt concentrations (0-0.9 %), when compared with their respective controls.

## Discussion

*Lantana montevidensis* leaf extracts have been used in traditional Brazilian medicine to treat various ailments including tumor and ulcer, in which free radicals have been implicated in their etiology (Demir et al., 2003[10]). Although *L. montevidensis* has been used for the same purpose as *L. camara*, there are however, very limited studies on its pharmacological activities as well as its safety. In this context, this study aimed at investigating the antioxidant capacity of *L. montevidensis* using various assays' systems, and its toxicity in human leukocytes and erythrocytes.

In biological system, antioxidants are extremely important due to their direct removal of pro-oxidants in order to ensure maximum protection for biological sites (Pisoschi and Pop, 2015[28]; Kamdem et al., 2016[18]; Hassan et al., 2017[16]). Iron(II) is a potent pro-oxidant that has been found to be increased in many neurodegenerative disorders associated with oxidative stress, including Parkinson's and Alzheimer's diseases (Zecca et al., 2008[40]; Becerril-Ortega et al., 2014[5]). Indeed, Fe^2+^ has been extensively used *in vitro* to access the antioxidant potential of chemicals or plant extracts, since it can induce oxidative stress via the generation of ROS (Harris et al., 1992[14]). In the current study, Fe^2+^ caused a significant increase in the amount of malondialdehyde (MDA), one of the most abundant by-products of lipid peroxidation (LPO) generated via radical-initiated oxidative decomposition of polyunsaturated fatty acid (Singh, 2015[31]). Interestingly, EtOH and aqueous extracts from the leaves of *L. montevidensis* efficiently inhibited Fe^2+^-induced LPO in rat brain and liver homogenates. These results suggest that the components of both extracts may have interacted with free radicals or chelated free iron, thereby, rendering it unavailable for the Fenton process. Our results are in agreement with that of Sousa et al. (2015[34]) who showed that leaf and root extracts of *L. montevidensis* inhibit Fe^2+^-induced LPO in egg phospholipids.

The biological activity of transition metals such as iron is associated with the presence of unpaired electrons that favour their participation in redox reaction (Repetto et al., 2010[29]). For instance, they can participate in the initiation step of LPO, DNA assault and oxidative stress through metal-catalyzed reactions (Aitken, 2014[1]; Hassan et al., 2017[16]). Here, we have investigated the Fe^2+^-chelating ability of EtOH and aqueous extracts of *L. montevidensis*. The results demonstrated that both extracts did not chelate Fe^2+^, suggesting that their antioxidant capacity observed against LPO seems to be associated with direct action with free radicals and not Fe^2+^ chelation. 

The free radical scavenging activity of EtOH and aqueous extracts was evaluated by the DPPH assay. In this assay, the purple color of the DPPH is changed to the yellow color in the presence of hydrogen donating antioxidant. Thus, the more yellowish color of DPPH observed, the greater is the antioxidant activity of the tested compound/extract. Both extracts caused a concentration-dependent reduction in the color of DPPH radical, suggesting their hydrogen donating ability. However, the aqueous extract exhibited a higher DPPH radical scavenging activity than the EtOH extract. The difference in this activity may be explained by the ability of the aqueous extract to quickly donate hydrogen atoms to the DPPH radical than the EtOH extract. It has been postulated that molecules that can donate hydrogen atoms to damaged molecules can be considered as repair compounds (Kohen and Nyska, 2002[21]). Thus, it is possible to extrapolate that the donation of hydrogen atoms by EtOH and aqueous extracts to fatty acid radical which was previously attacked by a radical (and lost its hydrogen) in the Fe^2+^-induced LPO, has contributed to limit the damages or inhibit LPO.

The exceptional growth of human exposure to natural products particularly from plants origin has led to the resurgence of the scientific interest in their biological activities, with emphasis on their toxicological evaluations in order to ascertain their safety (Kamdem et al., 2013[19]; Barbosa-Filho et al., 2014[2]; Waczuk et al., 2015[39]; Duarte et al., 2016[11]). Substantial evidences from literatures reveal that plants used in traditional medicine or as food can have mutagenic, cytotoxic and genotoxic effects *in* *vitro* and *in vivo *(Zink and Chaffin, 1998[41]; Celik, 2012[6]). Here, the effect of EtOH and aqueous extracts of *L. montevidensis* on DNA of human leukocytes was evaluated by the comet assay, which is regarded as a valuable biomarker of genotoxicity (Matić et al., 2013[24]). We found that both extracts (1-480 µg/mL) did not have genotoxic effect in relation to the control. However, at the higher concentrations tested (240-480 µg/mL), they showed a slight cytotoxic effect. Although we have not identified the constituent(s) responsible for the cytotoxic effect, some of the compounds identified includes rutin, quercetin and kaempferol, and these have been shown to have toxic effects at relatively high concentrations (Soares et al., 2006[33]). Consequently, it is possible to presume that the DNA damage evaluated by the Comet assay can occur in parallel or not with the toxicity detected as decreased survival (by Trypan blue). Despite the fact that it is difficult to translate the *in vitro* results obtained here to a real *in vivo* situation, our findings suggest that caution should be taken regarding the dosage and the frequency use of *L. montevidensis* in the traditional medicine.

Exposure of human erythrocytes to phyto-therapeutic products can influence erythrocyte membrane integrity making the cells more fragile and labile to damage (Mpiana et al., 2010[25]). In the present study, osmotic fragility was used as a useful cytotoxic indicator to evaluate the interactions of *L. montevidensis* extracts with the cell membrane *in vitro*. However, *L. montevidensis* extracts did not have any effect on the osmotic fragility of erythrocytes, suggesting that the antioxidant defense system of the erythrocytes may have contributed to protect the cell membranes. 

The chemical composition of* L. montevidensis* extracts showed the presence of polyphenolic compounds (phenolic acids and flavonoids). Their long-term consumption has been shown to be associated with reduced risk of cardiovascular diseases, neurodegenerative diseases, cancer and diabetes among others in epidemiological studies (Lall et al., 2015[22]). Therefore, the action of these phytochemicals alone or synergistically could be responsible for radical scavenging activity and the prevention of LPO observed in this study.

## Conclusions

Overall, the present study investigated for the first time, the toxicity of ethanolic and aqueous extracts from the leaves of *L. montevidensis* in human cells, and their antioxidant activity in various assay systems. Exposure of human leukocytes to higher concentrations of both extracts (240-480 µg/mL) caused cytotoxicity, without any sign of DNA damage, indicating that caution should be taken regarding the dosage and frequent use of *L. montevidensis* in traditional medicine. However, treatment of human erythrocytes with both extracts (1-480 µg/mL) did not have any effect on erythrocyte membranes. The ethanolic and aqueous extracts exhibited strong antioxidant activity as evidenced by their DPPH radical scavenging activity and their efficacy to inhibit lipid peroxidation in rat brain and liver homogenates. These effects can be attributed to the presence of bioactive phytochemicals found in *L. montevidensis*, and may therefore justify its pharmacological use in folk medicine.

## Conflict of interest

The authors declare no conflict of interest with any person or any organization.

## Acknowledgements

Dr. Luiz Marivando Barros is particularly grateful to the CAPES/DINTER/URCA-UFSM Program. Dr. Jean Paul Kamdem acknowledges the financial support of FUNCAP, CNPq, TWAS, and CNPq-TWAS.

## Figures and Tables

**Table 1:**
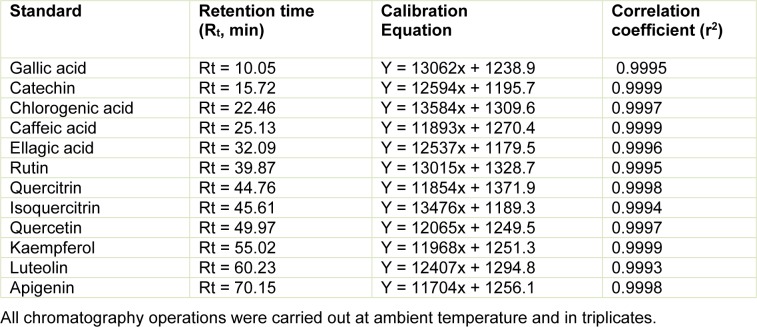
Calibration curve of the standards used in the analysis

**Table 2:**
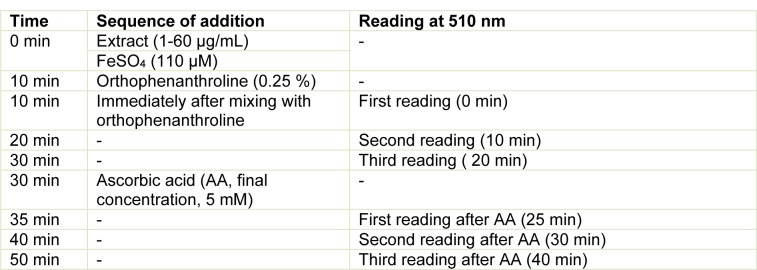
Schedule of evaluation of oxidation or chelation of Fe^2+^/Fe^3+^ by plant extracts

**Table 3:**
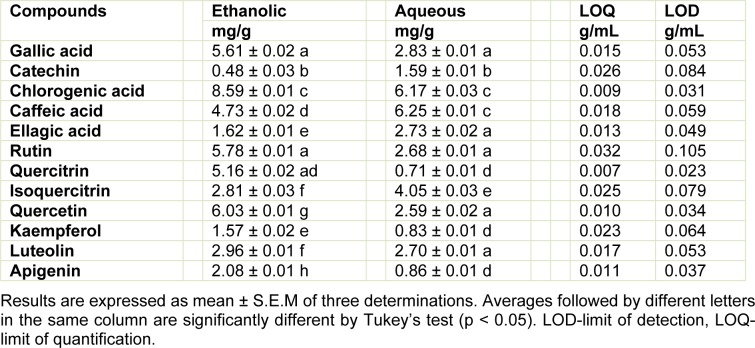
Quantitative analysis of phenolics and flavonoids from the ethanolic and aqueous extracts of *L. montevidensis *leaves

**Table 4:**
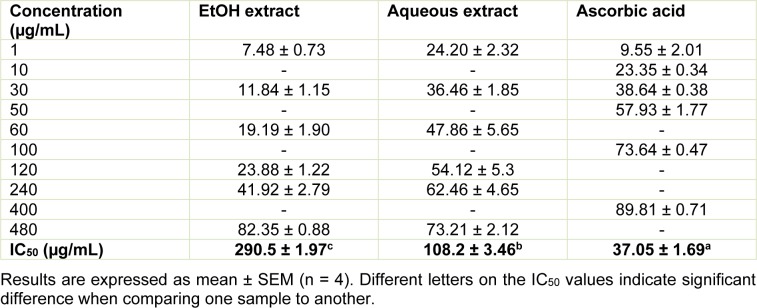
Percentage inhibition of DPPH radical by EtOH and aqueous extracts from the leaves of *L. montevidensis*
